# The Longitudinal Relationship Between Dark Triad Traits and Moral Disengagement in Adolescents: A Cross-Lagged Panel Network Analysis

**DOI:** 10.3390/bs16030398

**Published:** 2026-03-09

**Authors:** Huanhuan Zhao, Kaiwen Wang, Yan Xu, Heyun Zhang

**Affiliations:** 1 School of Psychology, Shanghai Normal University, Shanghai 200234, China; hhzhaopsy@shnu.edu.cn (H.Z.); 1000568671@smail.shnu.edu.cn (K.W.); 2 Faculty of Psychology, Beijing Normal University, Beijing 100875, China; xuyan@bnu.edu.cn

**Keywords:** Dark Triad traits, moral disengagement, cross-lagged panel network analysis, gender difference, adolescent

## Abstract

Moral disengagement (MD) typically peaks during adolescence. While the Dark Triad (DT) traits—Machiavellianism, psychopathy, and narcissism—are broadly linked to MD, the specific prospective pathways through which individual DT components predict distinct MD strategies remain unclear, particularly with respect to gender-specific variations in these influences among adolescents. To systematically investigate these temporal associations, this study employed Cross-Lagged Panel Network (CLPN) modeling on a sample of 1410 Chinese adolescents (*M*_age_ = 16.95, *SD* = 0.75) surveyed across three waves at three-month intervals. Results revealed a hierarchical pattern of DT influence: Machiavellianism exerted the strongest predictive effect on the MD system, followed by psychopathy, while narcissism showed negligible or even negative effects. Among MD strategies, euphemistic labelling, advantageous comparison and displacement of responsibility were the most responsive to DT traits. Bridge centrality analysis confirmed Machiavellianism as the primary cross-domain connector linking DT traits to MD. Weak but significant reciprocal effects were observed: MD slightly fed back onto later Machiavellianism and psychopathy, supporting a partially bidirectional process. Gender-separated networks revealed divergent pathways: Machiavellianism served as the key DT-MD bridge for males, whereas psychopathy fulfilled this role for females. These findings refine the understanding of the “dark side” of moral development by highlighting mechanism-specific MD vulnerabilities and demonstrating that the primary socio-cognitive pathway to MD is gender-contingent, thereby advancing developmental models of MD.

## 1. Introduction

Adolescence marks a crucial period characterized by significant shifts in moral development (Bajovic & Rizzo, 2021). During this phase, adolescents experience rapid advancements in moral reasoning (Lapsley & Carlo, 2014) alongside a relatively high incidence of immoral behavior (Tremblay, 2010). This confluence of trends renders them “both emboldened to transgress and adept at justifying”—often developing rationalizations when their actions conflict with internal moral standards (Sijtsema et al., 2019). The fundamental psychological process underlying this self-regulatory breakdown is moral disengagement (MD) (Bandura, 1999), which systematically rationalizes misconduct via cognitive restructuring. Given that MD levels typically peak during adolescence (Paciello et al., 2008) and strongly predict aggressive and antisocial outcomes (e.g., Hyde et al., 2010; Paciello et al., 2008; Sijtsema et al., 2019), understanding its etiology—particularly how predisposing personality factors such as the Dark Triad (DT) shape its development—is essential for both theoretical advancement and the development of effective prevention strategies. However, extant research has predominantly relied on aggregate variable-centered approaches that treat MD as a unitary construct, leaving the specific trait-cognition correspondences and their reciprocal dynamics over time poorly understood. To bridge this gap, the present study adopts a dimension-specific, process-oriented perspective, employing Cross-Lagged Panel Network (CLPN) analysis to examine the granular, longitudinal interplay between specific DT traits and MD strategies in Chinese adolescents, and to ascertain whether these pathways operate differentially across gender.

### 1.1. Dark Triad Traits and Moral Disengagement

Personality traits are fundamental to MD and its link to immoral conduct (Egan et al., 2015). Research confirms a positive association between personality known as the DT traits (Paulhus & Williams, 2002) and MD (e.g., Egan et al., 2015; Y. Li et al., 2026; Sijtsema et al., 2019), with individuals high in DT traits more prone to MD. Importantly, this relationship may be bidirectional: DT traits drive MD strategy use, and repeated MD may reinforce DT traits, forming a feedback loop that warrants further investigation.

The DT comprises three distinct sub-personality traits—Machiavellianism, Narcissism, and Psychopathy—that share maladaptive characteristics but differ in motivational tendencies (Jonason et al., 2015). Machiavellianism centers on instrumental manipulation and interpersonal exploitation, driven by a utilitarian orientation toward self-interest. As strategic and context-flexible planners (D. N. Jones & Paulhus, 2009, 2011), Machiavellians rely on a domain-general cognitive repertoire that supports the coordinated deployment of self-serving cognitive strategies. Narcissism is marked by grandiosity, an excessive need for admiration and self-aggrandizement, and a self-enhancement motivational tendency, alongside relatively high residual empathy (Jonason & Kroll, 2015). Psychopathy is defined by a callous and impulsive dispositional inclination, manifested in emotional and interpersonal coldness, a lack of remorse, and impulsive tendencies (Paulhus & Williams, 2002; Aharoni et al., 2014).

In parallel, MD is a cognitive construct consisting of eight distinct strategies that justify un-ethical acts (Bandura, 1999), serving as operational tools for enacting DT motivations: Moral justification (framing misconduct as socially acceptable), Advantageous comparison (minimizing wrongdoing by contrasting it with worse behaviors), Euphemistic labeling (using sanitized language), Displacement of responsibility (attributing actions to external pressures), Diffusion of responsibility (diluting personal accountability in group settings), Distortion of consequences (ignoring or minimizing harm), Dehumanization (stripping victims of their human qualities), and Attribution of blame (shifting responsibility onto the victim). Different MD strategies exert varied influences on behavioral outcomes (Bandura, 1999). This differentiation between DT’s motivational traits and MD’s cognitive strategies underscores the need to explore specific trait-strategy correspondences.

To date, while DT-MD associations are well-documented (e.g., Egan et al., 2015; Y. Li et al., 2026; Sijtsema et al., 2019), two critical gaps remain: First, reliance on aggregated MD scores masks crucial trait-strategy heterogeneity. For instance, Y. Li et al. (2026) found that Machiavellianism and psychopathy are more strongly associated with overall MD than narcissism, with this association persisting over time while narcissism shows no significant link. These findings suggest different DT traits exert heterogeneous impacts, likely linking to distinct MD strategies. Existing research, however, relies on holistic models that aggregate MD strategies into a single score, obscuring specific trait-strategy associations, conflating distinct mechanisms, and hindering precise theoretical specification.

Second, the pervasive use of cross-sectional designs cannot capture longitudinal associations, including directionality and reciprocal feedback loops critical for understanding personality development. The specific temporal processes linking these constructs remain unclear, obscuring the intricate underlying dynamics of adolescent MD. Although some longitudinal studies exist (e.g., Y. Li et al., 2026; Sijtsema et al., 2019), they still treat MD as a unitary construct and do not analyze DT and MD at the dimensional level. For example, Sijtsema et al. (2019) only examined total-score predictions rather than specific trait–strategy dynamics, limiting theoretical and practical value. Traditional cross-lagged models cannot capture this complexity, indicating a need for a finer-grained longitudinal approach.

### 1.2. Gender Differences

Gender differences should be considered when examining the link between DT and MD (K. E. Moore et al., 2020), given clear gender differences in both constructs. Males generally score higher on MD (Detert et al., 2008; Gómez-Tabares, 2024; Hyde et al., 2010; Paciello et al., 2008), possibly due to differences in moral norm internalization during socialization (Thornberg & Jungert, 2014). DT traits also differ substantially by gender: males often score higher overall (Goncalves & Campbell, 2014; Jonason & Webster, 2010; Rogoza et al., 2021), and male and female adolescents differ in how DT traits are organized and expressed (Jonason et al., 2013; D. N. Jones & de Roos, 2017; D. N. Jones & Weiser, 2014). This divergence suggests the developmental pathways connecting DT and MD may differ by gender. Males tend to use more externalizing moral justification strategies, while females rely more on internalizing or affective detachment (Gómez-Tabares, 2024; Rubio-Garay et al., 2019). Thus, the associations between specific DT traits and MD strategies may be gender-contingent.

Accordingly, this study asks whether gender heterogeneity exists in the longitudinal network structure linking DT and MD. Despite strong theoretical support for gender-specific patterns, existing research has not examined the dynamic, dimensional-level DT–MD relationship from a gender-specific perspective.

### 1.3. Network Analysis and Theory

To address these gaps, we use CLPN analysis—an approach ideal for dimensional, longitudinal investigations into dynamic psychological processes. Network analysis models psychological constructs as interactions between nodes (specific DT facets, distinct MD strategies) and edges (their associations), rather than treating them as unitary latent variables (Fried et al., 2017; Epskamp et al., 2012). CLPN overcomes prior limitations by: (a) modeling dimension-specific DT-MD associations that traditional aggregate analyses overlook, (b) capturing bidirectional temporal dynamics and feedback loops, and (c) identifying bridging nodes linking DT and MD communities.

Three key centrality metrics clarify node influence in the DT-MD network: Out Expected Influence (O-EI) quantifies a node’s predictive capacity—high O-EI for a DT trait signals strong prediction of subsequent MD strategy changes (identifying “initiators” of MD); In Expected Influence (I-EI) measures received influence, with high I-EI for an MD strategy indicating vulnerability to DT trait impacts; and Bridge Expected Influence (B-EI) captures cross-community connectivity (Heeren et al., 2018; Rhemtulla et al., 2017), with high B-EI nodes acting as critical “bridges” mediating DT-MD activation and identifying intervention targets. CLPN leverages these metrics across three time points to capture developmental trajectories, revealing dynamic relationships undetectable by traditional methods.

### 1.4. Current Study

Existing research has hitherto failed to adequately capture the nuanced features and dynamic, dimension-specific temporal associations between DT and adolescent MD. The CLPN analysis offers a novel, systematic framework for longitudinal exploration, one that, to our knowledge, has not been systematically applied to DT and MD. Therefore, the primary goal of this study is to utilize CLPN analysis to delineate the fine-grained, longitudinal network between DT traits and MD strategies in Chinese adolescents, identifying core predictors (high O-EI), primary targets (high I-EI), and crucial bridges (high B-EI) that facilitate the DT-MD linkage. Furthermore, the second aim of this study is to test whether the aforementioned longitudinal network structure is moderated by gender, by constructing and comparing separate CLPNs for males and females, thereby uncovering potential gender-specific pathways from DT to MD. This comparative analysis addresses potential gender heterogeneity in the DT-MD relationship, filling the gap in existing research regarding gender-specific dynamic associations.

## 2. Methods

### 2.1. Participants and Procedure

Participants were recruited through convenience sampling from secondary vocational schools in Zhejiang, China, following approval from the Academic Ethics Committee of the researcher’s university. Data were collected across three waves at three-month intervals after obtaining informed consent from adolescents, their parents, and teachers. Participants completed a confidential web-based survey in computer-equipped classrooms. To support complete data collection for longitudinal analyses, the survey system guided participants to review any unanswered items before submission. Participants were fully informed that they could withdraw or discontinue their participation at any time without penalty. They were thoroughly briefed on the research purpose and procedures, with emphasis on confidentiality to mitigate social desirability bias. All participants received appropriate compensation for their involvement.

At Time 1 (T1; March 2023), 1909 adolescents (52.70% males; age range: 15~20 years; *M*_age_ = 16.68 years, *SD* = 0.83 years) participated in the survey, providing data on demographics, DT traits, and MD. In terms of parental education, most parents had a junior high school education or below (fathers: 59.50%; mothers: 61.00%), while fewer had a senior high school education (fathers: 31.10%; mothers: 29.60%), and the smallest proportion held a bachelor’s degree or higher (fathers: 9.50%; mothers: 9.30%). Regarding family monthly income, 28.70% of families reported monthly incomes below 5000 CNY, a plurality (48.20%) earned between 5001 and 10,000 CNY, 17.10% earned between 10,001 and 20,000 CNY, and 6.00% earned above 20,001 CNY. At Time 2 (T2; June 2023), 1674 adolescents from the initial T1 sample continued participation. The primary reason for attrition was that 235 adolescents were absent on the assessment day due to off-campus internships or school transfers at the end of the academic semester. At Time 3 (T3; September 2023), 1430 adolescents from the T2 sample were retained, while 244 were lost to follow-up due to graduation or off-campus internships after T2. Attrition analysis revealed no significant differences at T1 between adolescents who completed all three waves and those who dropped out at T2 and/or T3 on gender (χ^2^(1) = 0.99, *p* = 0.32), age (*t* = −1.63, *p* = 0.10), DT traits (*t* = −1.51, *p* = 0.13), or MD (*t* = −1.52, *p* = 0.13), suggesting that attrition did not substantially bias the data. The final valid longitudinal data set consisted of 1410 participants (53.60% males; age range: 15–20 years; *M*_age_ = 16.95 years, *SD* = 0.75 years), with an additional 20 responses excluded for failing to meet rigorous response standards (e.g., patterned responding, failure to pass two deception detection questions).

### 2.2. Measures

#### 2.2.1. Dark Triad Traits

This study used the 12-item Dirty Dozen scale (Jonason & Webster, 2010) to assess the dark personality traits of adolescents. The scale consists of three subscales: Machiavellianism, Psychopathy, and Narcissism. Participants responded to the items using a 7-point Likert scale, ranging from 1 (strongly disagree) to 7 (strongly agree). This brief scale was selected for its confirmed cross-cultural structural invariance across eight world regions, including Asia (Rogoza et al., 2021), and its wide application and validation in Chinese adolescent populations (Shen, 2023). In this study, the Cronbach’s α at the three time points were 0.88, 0.91, and 0.90, respectively.

#### 2.2.2. Moral Disengagement

This study used a culturally adapted Chinese version of the 8-item MD scale (Detert et al., 2008; C. Moore et al., 2012). The scale was translated and back-translated to ensure linguistic and conceptual equivalence with the original measure, with minor wording adjustments to improve comprehension for Chinese adolescents. The questionnaire measures eight distinct MD mechanisms: moral justification, euphemistic labeling, advantageous comparison, displacement of responsibility, diffusion of responsibility, distortion of consequences, attribution of blame, and dehumanization. Participants responded using a 7-point Likert scale ranging from 1 (strongly disagree) to 7 (strongly agree). This adapted scale has been widely used and validated among Chinese adolescent samples (Zhao et al., 2025). In this study, the Cronbach’s α at the three time points were 0.80, 0.89, and 0.88, respectively.

### 2.3. Data Analysis

Statistical analyses were conducted in R (Version 4.2.0). Listwise deletion was used to handle missing data, with only participants who provided complete data across all three time points (T1, T2, T3) for all DT traits and MD strategies included in the CLPN analyses. The CLPN model used the glmnet package for regularized regression (LASSO penalty) to derive autoregressive and cross-lagged coefficients, optimized via 10-fold cross-validation for sparsity. The resulting networks were visualized using the graph package. Edge interpretation was standardized: blue edges represented positive associations and red edges negative ones. The thickness of the edges represents the strength of the relationship, with thicker edges indicating stronger relationships. To reduce visual clutter and improve the interpretability of the focal cross-domain relationships, intra-cluster edges (i.e., predictive relations within the DT trait cluster or within the MD strategy cluster) and cross-lagged edges within the same construct community were omitted from the network visualizations. Critically, to permit direct visual comparison across different time points, we enforced a consistent layout using the qgraph package’s average Layout function, ensuring all nodes maintained fixed positions (Epskamp & Fried, 2018). The I-EI and O-EI were estimated to assess directional predictive relationships between DT traits and MD strategies, and B-EI to identify bridging items between the two domains.

Network stability and accuracy were evaluated using the bootnet package. Stability was assessed via non-parametric bootstrapping (1000 samples) to generate 95% Confidence Intervals (CIs); narrow CIs confirm stable edges (Epskamp & Fried, 2018). Network parameter accuracy was verified using the Correlation Stability (CS) coefficient. A CS value above 0.25 (ideally > 0.05) confirms robustness, indicating that excluding a small proportion of samples does not significantly alter centrality metrics (Epskamp & Fried, 2018). Gender-specific network comparisons were conducted by assessing edge list similarity, the cumulative percentage of overlapping associations, and the Jaccard similarity index.

## 3. Results

### 3.1. Preliminary Analysis

Table 1 presents item information, node labels, means, and standard deviations for DT traits and MD at three time points.

### 3.2. Network Analysis

**Figure 1:**
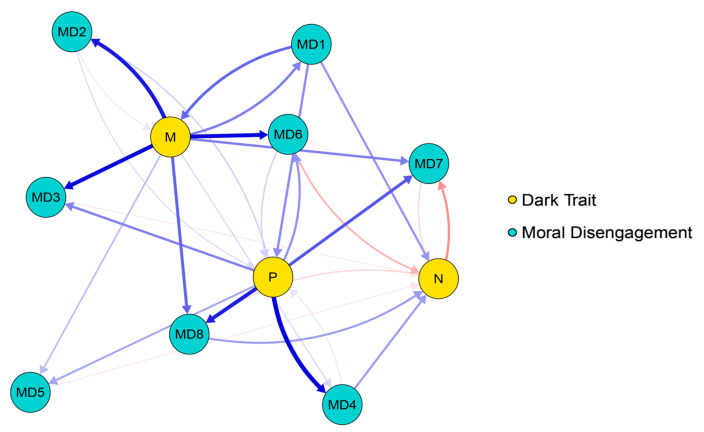
Cross-lagged network between T1 and T2 time periods. Note. The arrows represent unique longitudinal relationships, with blue edges indicating positive relationships and red edges indicating negative relationships. The thickness of the edges represents the strength of the relationship, with thicker edges indicating stronger relationships.

**Figure 2:**
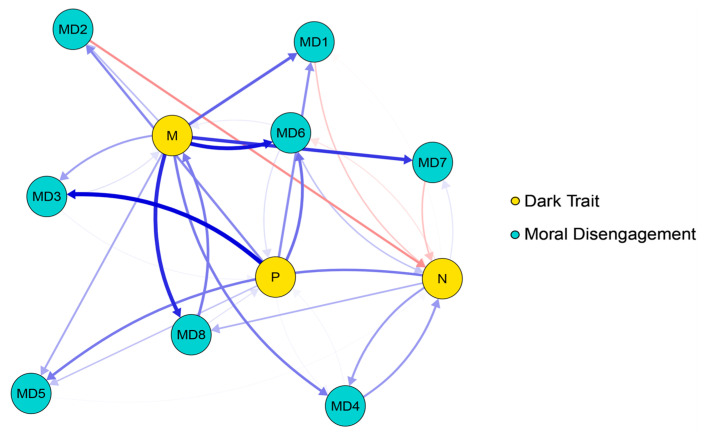
Cross-lagged network between the T2 and T3 time periods. Note. The arrows represent unique longitudinal relationships, with blue edges indicating positive relationships and red edges indicating negative relationships. The thickness of the edges represents the strength of the relationship, with thicker edges indicating stronger relationships.

The cross-lagged networks of DT and MD are presented in Figure 1 and Figure 2. The edge weight matrices for the two cross-lagged networks are provided in Supplementary Materials Tables S1 and S2. In the T1–T2 network, there are 69 non-zero edges (62%), while in the T2–T3 network, there are 71 non-zero edges (64%). At the T1–T2 time point, the edges with the strongest cross-lagged effects are: M–MD2, M–MD3, M–MD6; P–MD4, P–MD8. At the T2–T3 time point, the edges with the strongest cross-lagged effects are: M–MD1, M–MD6, M–MD7, M–MD8; P–MD3, P–MD6. The strongest connecting edge at the T1–T2 time point is P–MD4, while the strongest connecting edge at the T2–T3 time point is P–MD3.

### 3.3. Centrality

**Figure 3:**
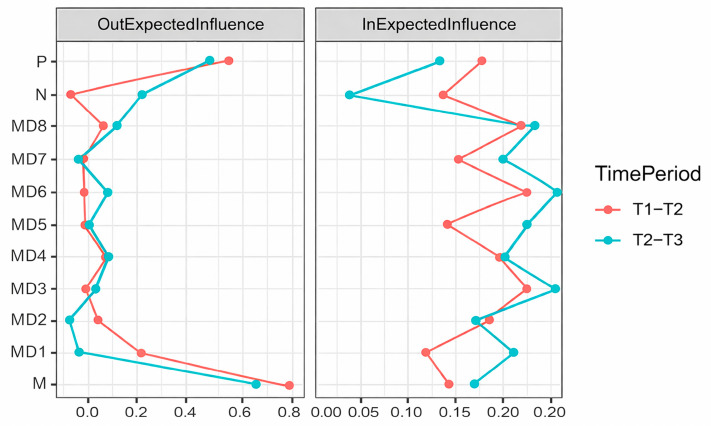
Estimation of node centrality in the T1–T2 and T2–T3 cross-lagged networks. Note. Red represents the T1–T2 period, and blue represents the T2–T3 period. The X-axis represents the value of the centrality index, with larger values indicating greater bridge centrality.

The network centralities of DT and MD are shown in Figure 3. The DT community has a higher O-EI, while the MD community has a higher I-EI, indicating that in this network, DT are the initiators of influence, while the MD community plays the role of being influenced. Regarding DT, Machiavellianism has the highest O-EI at both time points, meaning it significantly predicts other symptoms. Psychopathy has the second-highest influence, while narcissism has a lower influence at both time points, with even some negative influences, especially on MD6 (Distortion of Consequences) and MD7 (Attribution of Blame) at the T1–T2 period. MD2, MD3, and MD4 have the highest I-EI, indicating that these three nodes are most influenced in the network. MD has a much smaller impact on DT traits than vice versa; among these, psychopathy and Machiavellianism are more affected than narcissism, since their I-EI is bigger than narcissism in both time periods.

**Figure 4:**
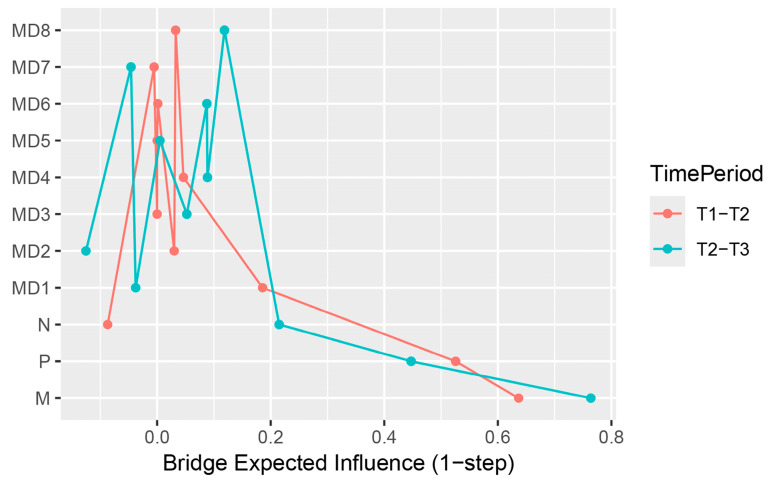
Estimation of node bridge centrality in the T1–T2 and T2–T3 cross-lagged networks. Note. Red represents the T1–T2 period, and blue represents the T2–T3 period. The X-axis represents the value of the centrality index, with larger values indicating greater bridge centrality.

The bridge centrality estimation shows the degree to which nodes link the two communities, and the results are shown in Figure 4. Machiavellianism has the highest B-EI at both time points, with psychopathy having the second-highest B-EI, and narcissism having a relatively small B-EI. This means that Machiavellianism has the strongest predictive effect on MD, followed by psychopathy, with narcissism having the weakest predictive effect.

### 3.4. Stability Analysis

The confidence intervals of the edge weights for the cross-lagged networks at both time points are narrow (see Supplementary Materials Figures S1 and S2), with more overlap between the 95% confidence intervals of the edge weights. Some of the strongest edges do not overlap with the confidence intervals, indicating that these edges are accurate. The bootstrap results (see Supplementary Materials Figures S3 and S4) show that the stability of O-EI (both CS coefficients = 0.52), I-EI (CS coefficients = 0.52 and 0.67), and B-EI (both CS coefficients = 0.59) is all good.

### 3.5. Gender-Based Network Comparison

**Figure 5:**
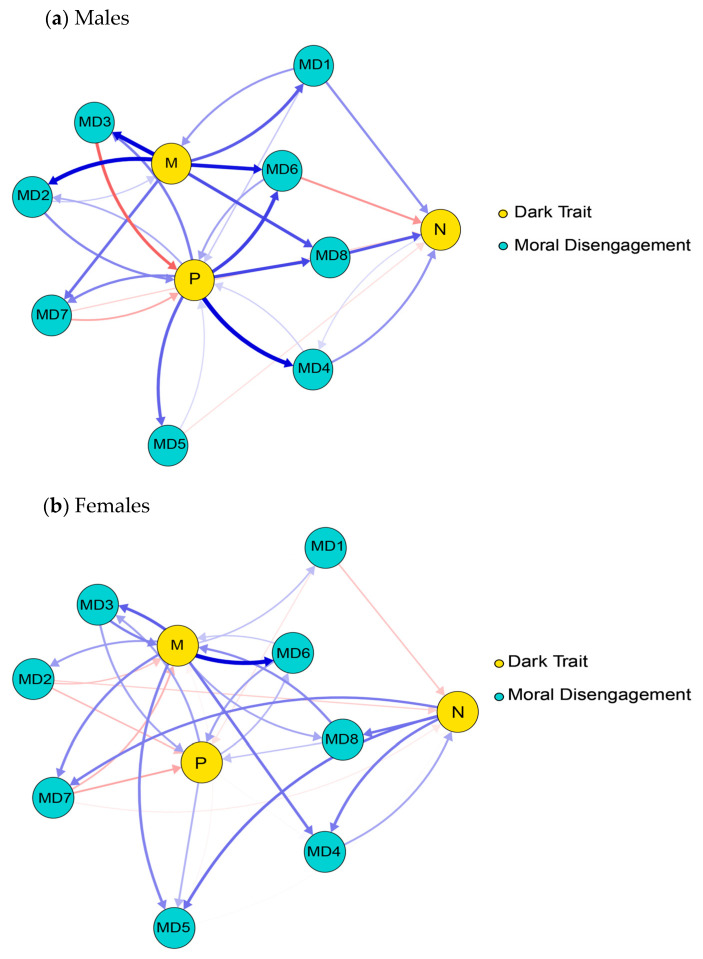
The cross-lagged network for males (**a**) and females (**b**) between the T1 and T2 time periods. Note. The arrows represent unique longitudinal relationships, with blue edges indicating positive relationships and red edges indicating negative relationships. The thickness of the edges represents the strength of the relationship, with thicker edges indicating stronger relationships.

**Figure 6:**
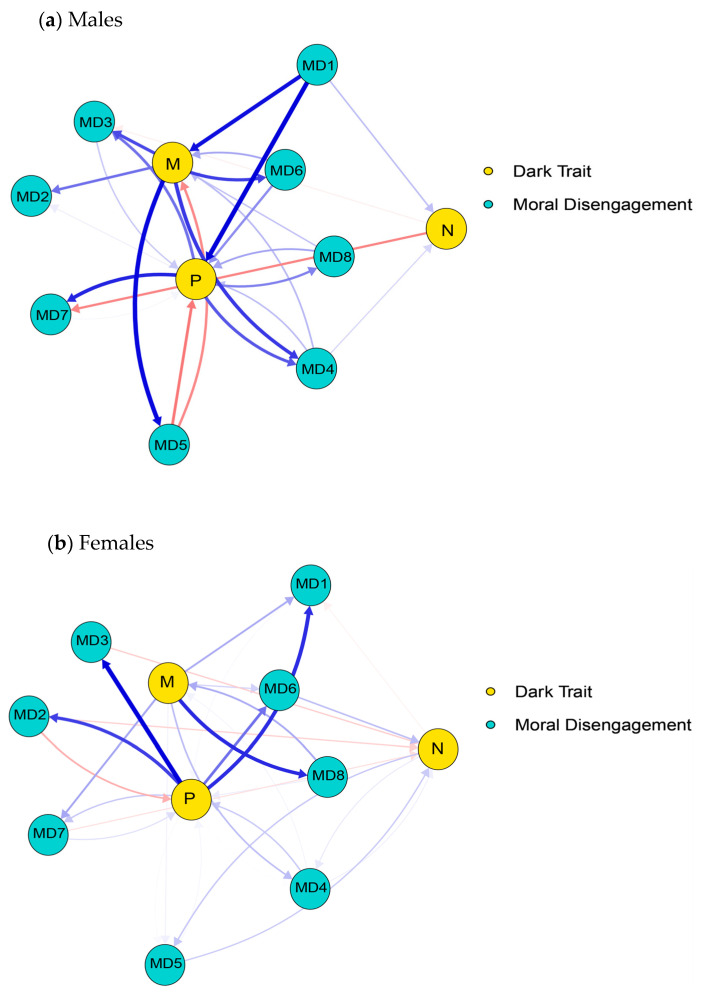
The cross-lagged network for males (**a**) and females (**b**) between the T2 and T3 time periods. Note. The arrows represent unique longitudinal relationships, with blue edges indicating positive relationships and red edges indicating negative relationships. The thickness of the edges represents the strength of the relationship, with thicker edges indicating stronger relationships.

We compared the networks for males and females at three time points to examine whether there are gender differences in the relationship between DT traits and MD. For cross-lagged network graphs of different genders in different time periods, as shown in Figure 5 and Figure 6. The comparison results show that the correlation between edge lists for gender networks is moderate to low (*r*_T1–T2_ = 0.42, *r*_T2–T3_ = 0.40), suggesting that most of the edge connection patterns differ by gender. The Jaccard similarity index further confirms that while there is some overlap in the gender networks (T1–T2 = 0.46, T2–T3 = 0.42), the overall structure differs significantly.

**Figure 7:**
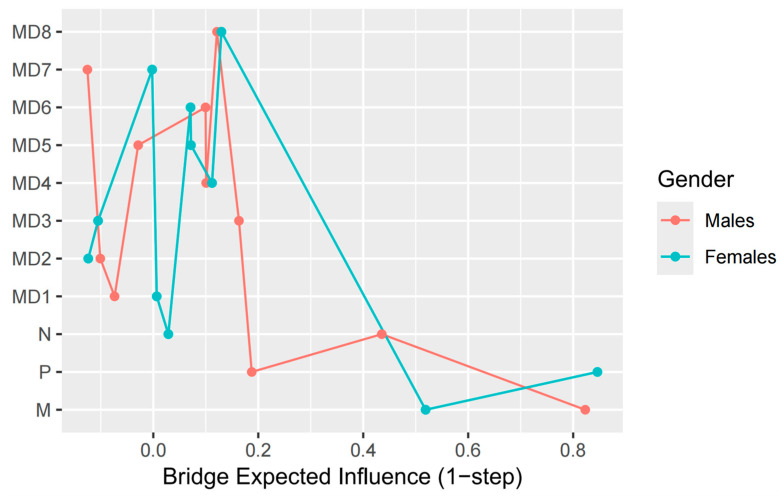
Estimation of bridge centrality of nodes in the T2–T3 cross-lagged network for different genders. Note. Red represents males, and blue represents females. The X-axis represents the value of the bridge centrality index, with larger values indicating greater bridge centrality.

Since the case-drop bootstrap showed that the stability of the in-strength can be poor (see Supplementary Materials Figures S6–S8, CS coefficient_male T1–T2_ = 0.18; CS coefficient_male T2–T3_ =0.28; CS coefficient_female T1–T2_ = 0.28; CS coefficient_female T2–T3_ = 0.28), we focused only on the bridge centrality of the T2–T3 period (CS coefficient_maleT1–T2_ = 0.44; CS coefficient_maleT2–T3_ = 0.36; CS coefficient_female T1–T2_ = 0.21; CS coefficient_female T2–T3_ = 0.52). In the T2–T3 period (Figure 7), Machiavellianism has the highest bridge-EI in the male network (B-EI = 0.82), whereas psychopathy has the highest bridge-EI in the female network (B-EI = 0.80).

## 4. Discussion

The longitudinal architecture, mechanism-specific pathways, and potential moderators underlying the relationship between DT and MD remain under-explored, despite the general association being well documented. The present study is the first to employ CLPN analysis to dissect the bidirectional, time-lagged relations among the DT traits and eight distinct MD strategies across three waves in Chinese adolescents, while simultaneously testing for gender-differentiated network wiring. These findings effectively advance the field beyond a generic “DT predicts MD” statement toward a high-resolution, mechanism-specific, and gender-contingent model. The results demonstrate that DT effects on moral cognition are heterogeneous: Machiavellianism functions as a broadband predictor, psychopathy acts as a precision mechanism driver, and narcissism operates as an inhibitory node. Furthermore, moral cognition exhibits reciprocal influence, nourishing Machiavellianism and psychopathy, yet actively eroding narcissism.

### 4.1. Relations Between DT Traits and MD

Machiavellianism emerged as the primary driver of MD across both time windows (T1→T2 and T2→T3), showing the highest O-EI and B-EI, which indicates its central role in predicting other network variables and connecting DT and MD communities. Critically, its predictive scope broadened over time: early predictions focused on euphemistic labeling (MD2), advantageous comparison (MD3), and distortion of consequences (MD6), while later predictions expanded to moral justification (MD1), attribution of blame (MD7), and dehumanization (MD8). This expansion aligns with Machiavellianism’s core psychological profile. Characterized by diminished self-awareness and reduced self-reflection, Machiavellians lack critical insight into the immoral nature of their conduct (Paulhus & Williams, 2002). Moreover, they tend to view others as mere instruments for self-serving goals, rather than as autonomous agents with distinct thoughts and emotions (D. N. Jones & Paulhus, 2011). Such dehumanizing tendencies enable them to disregard others’ feelings and rights when engaging in unethical acts, thereby further facilitating MD. As flexible strategic planners adapting to diverse social contexts (D. N. Jones & Paulhus, 2009, 2011), Machiavellians employ a domain-general cognitive toolkit rather than depending on fragmented or isolated strategies. This explains why Machiavellianism consistently surpasses psychopathy and narcissism in both network centrality and predictive magnitude.

Psychopathy exhibited the second-highest O-EI and B-EI, but its predictive trajectory was more circumscribed and appeared to be developmentally modulated. Early-stage psychopathy forecasted externalizing strategies—displacement of responsibility (MD4), diffusion of responsibility (MD5), and dehumanization (MD8)—whereas later-stage psychopathy shifted toward internalizing tactics: moral justification (MD1), euphemistic labeling (MD2), and advantageous comparison (MD3). The strongest cross-lagged edge shifted from P→MD4 (T1–T2) to P→MD3 (T2–T3), reflecting a transition from impulsive blame-shifting to calculated cognitive reframing. This shift aligns with adolescent cognitive development theories (Gibbs, 2014): as adolescents typically improve in executive control and abstract reasoning, those high in psychopathy may move from rudimentary externalizing behaviors to sophisticated semantic restructuring to manage moral conflict. Trait-wise, psychopathy’s inherent low empathy (Ritchie & Forth, 2016) and interpersonal callousness (Aharoni et al., 2014) reduce sensitivity to others’ suffering, prompting moral distress relief via distorted interpretations of behavioral outcomes. Cognitively, advancing moral reasoning tends to replace emotionally driven externalizing tactics with cognitively elaborated moral restructuring. Thus, the observed longitudinal network patterns illustrate what appears to be the dynamic interplay between dispositional traits and age-typical cognitive development in the formation of MD.

Narcissism exhibited the lowest O-EI and B-EI and even displayed negative cross-lagged weights to several MD nodes—especially distortion of consequences (MD6) and attribution of blame (MD7) during T1–T2. Compared with Machiavellianism and psychopathy, narcissism is characterized by greater residual empathy (Jonason & Kroll, 2015) and higher self-efficacy (Petrides et al., 2011). Empathetic individuals tend to sympathize with victims and adopt compensatory behaviors instead of using MD to avoid responsibility (Detert et al., 2008). Because distortion of consequences and attribution of blame rely on downplaying harm or blaming victims, they require suppressing empathy for victims’ suffering, which narcissistic adolescents are less likely to do. Meanwhile, high self-efficacy supplies the motivational and emotional resources to remedy transgressions rather than evade accountability (Bandura, 1989), strengthening self-sanctioning and reducing reliance on MD. These findings highlight the heterogeneity within the Dark Triad and warn against treating its three components as morally equivalent. Notably, the negative cross-lagged associations only apply to specific MD strategies; narcissists may still act self-servingly but use alternative self-image-preserving mechanisms (Campbell et al., 2004) rather than MD.

Centrality indices revealed a modest but significant reverse pathway from MD to the DT. Notably, the strength of influence from MD to DT was much smaller than the reverse, indicating a clear asymmetry in magnitude. Despite this modest effect size, Machiavellianism and psychopathy exhibited higher I-EI than narcissism, suggesting that MD can still exert a positive influence on these two traits, while potentially suppressing narcissism. As adolescent personality is still developing, frequent use of MD strategies may yield gradual environmental benefits (Lingán-Huamán et al., 2024; Tong et al., 2024) that reinforce maladaptive patterns and consolidate exploitative traits such as Machiavellianism and psychopathy. In contrast, narcissism centers on maintaining superiority and grandiosity (Campbell et al., 2006) by upholding a perfect self-image and denying flaws (Grijalva & Zhang, 2016). Because MD involves cognitive restructuring to rationalize immoral behavior, it implicitly acknowledges behavioral shortcomings, which undermines the “absolute correctness” required for narcissism and thus produces a suppressive effect. This magnitude disparity is theoretically meaningful: the dominant DT→MD path supports personality as a stable antecedent of moral cognition, while the weaker but significant MD→DT path indicates that moral cognition is not merely a passive product but can also provide cumulative feedback on personality. These asymmetrical feedback loops confirm that moral cognition and personality form a dynamic, reciprocal system, even though personality represents the more dominant driving force during adolescence.

### 4.2. Gender Comparisons in the Network

Incorporating a gender perspective is crucial when examining the longitudinal relationship between DT traits and MD. Moral cognitive development often follows gender-specific pathways (Gilligan, 1993), meaning that different genders may form distinct psychological mechanisms when confronting moral dilemmas. However, existing research has overlooked gender-specific expressions of personality traits—a gap that may lead to a one-sided understanding of MD, particularly in explaining gendered MD behavioral patterns among adolescents. Accordingly, the second aim of this study was to explore gender differences in this longitudinal influence.

The present study revealed clear gender heterogeneity in the DT-MD longitudinal network: male and female networks showed significant structural divergence, with moderate-to-low edge correlations and low Jaccard similarity indices confirming distinct connection patterns. However, stability analyses warrant cautious interpretation. Case-dropping bootstrap indicated that in-strength centrality in gender-specific networks showed only modest stability in certain periods. Accordingly, conclusions about the relative ranking of specific MD strategies within each gender should be interpreted as preliminary structural tendencies rather than definitive hierarchies. In contrast, bridge centrality demonstrated acceptable stability, leading us to focus on the T2–T3 bridge structure. Results showed that Machiavellianism displayed the highest B-EI in the male network, whereas psychopathy exhibited the highest B-EI in the female network. These findings suggest that Machiavellianism acts as the primary DT-MD bridge among males, while psychopathy serves this critical socio-cognitive function among females.

The observed gender differences in the core DT–MD bridging nodes may reflect gender-specific adaptive strategies in adolescents facing moral dilemmas. Males tend to focus on controlling the external environment and others’ responses to deflect moral responsibility and reduce internal conflict (Gómez-Tabares, 2024; Rubio-Garay et al., 2019)—reflecting externalizing problems (Gutman & Codiroli McMaster, 2020), a pattern that aligns with the core features of Machiavellianism (interpersonal manipulation and external control) as the primary male DT-MD bridge. In contrast, females rely more on internal emotional regulation and detachment to reduce empathic responses and avoid moral conflict (Gómez-Tabares, 2024)—reflecting internalizing problems (Gutman & Codiroli McMaster, 2020), which corresponds to psychopathy’s core traits of callousness and emotional detachment as the key female DT-MD bridge.

These divergent gendered adaptive strategies may be rooted in societal gender expectations and socialization processes (Chen et al., 2014; X. Li, 2017). During the socialization process, males are typically socialized to embody independence, decisiveness, and control over the external environment, and society often tends to tolerate relatively more aggressive behaviors (X. Li, 2017)—norms that may help establish Machiavellianism as the primary DT–MD bridging trait among males. Females, conversely, are expected to prioritize others’ feelings and strictly adhere to social norms (Chen et al., 2014), making overt external manipulation less socially permissible. The coldness and emotional detachment inherent in psychopathy may therefore serve as a self-protective mechanism within these traditional gender roles, enabling females to avoid expressing overt discomfort or conflict when confronted with moral challenges. Collectively, these accounts suggest the observed DT-MD association is achieved through potentially culturally scaffolded gender-specific pathways that uniquely exploit others (Machiavellianism in males) or anaesthetize the self (psychopathy in females), though this socialization-based explanation requires direct empirical testing in future research.

### 4.3. Limitations and Implications

This study has several limitations. First, the relatively short follow-up period, while useful for capturing short-term dynamics, may not reflect long-term developmental trajectories. Future studies with longer follow-ups are needed to characterize the long-term interplay between DT traits and MD. Second, self-report measures may introduce social desirability bias, especially for sensitive constructs such as callousness and unethical behavior. Adolescents may underreport morally controversial behaviors or cognitive strategies to maintain a positive image, potentially distorting the frequency and intensity of MD. Although self-report is widely validated in adolescent research and strict confidentiality was applied here, future work should use multi-informant and multi-method approaches (e.g., peer ratings) to reduce bias and improve measurement quality. Third, the DT scale used only assesses grandiose narcissism and does not capture vulnerable narcissism (Miller et al., 2011). Given that these subtypes may operate through distinct motivational and affective pathways to MD, future research using multidimensional narcissism scales is needed to clarify divergent links with MD strategies. Fourth, each MD mechanism was assessed with a single item, precluding reliability estimation. Although the 8-item MD scale (Detert et al., 2008; C. Moore et al., 2012) showed strong internal consistency at each wave, unknown single-item reliability may reduce precision in cross-lagged analyses. Future studies should employ multi-item scales for each MD strategy to improve reliability and strengthen validity. Finally, although overall edge-weight confidence intervals were generally narrow and key centrality indices showed acceptable stability, the case-dropping bootstrap indicated that in-strength centrality in some gender-specific networks fell below recommended thresholds. This implies that node rankings from subgroup in-strength analyses may be sensitive to sampling variability and should be interpreted cautiously. The lower stability likely stems from smaller gender-stratified sample sizes and the regularization inherent in CLPN estimation, which can increase variability in sparse networks. Future studies with larger samples and independent replication are needed to verify the robustness of gender-differentiated centrality patterns.

Notwithstanding these caveats, the study demonstrates that the DT→MD influence is not a monolithic process. Specifically, Machiavellianism provides a broadband pathway into diverse MD strategies; psychopathy offers a precision pathway, and narcissism appears to act as an inhibitor. Furthermore, MD reciprocally nourishes Machiavellianism and psychopathy while concurrently eroding narcissism—all within a system rewired by gender. Clarifying these heterogeneous, reciprocal, and gender-contingent pathways is theoretically important for moving the field beyond a simple, aggregated “DT predicts MD” framework toward a more precise, mechanism-based understanding of moral personality development. Practically, this knowledge is critical because adolescence represents a formative period in which both dark personality traits and moral cognitive styles are still malleable. By identifying which traits function as core hubs, which MD strategies are most strongly regulated, and how these pathways differ between males and females, the current CLPN findings provide a high-resolution map for designing targeted, mechanism-informed, and gender-tailored interventions aimed at interrupting the maladaptive developmental cascade from dark personality traits to MD. In this way, the present findings contribute not only to theoretical advancement but also to meaningful implications for adolescent ethical education and prevention practice.

## 5. Conclusions

This study employs CLPN analysis to explore the longitudinal relationship between DT traits and MD among adolescents, with a focus on gender differences. Key findings reveal that DT traits are primary influencers, while MD is mainly influenced. Machiavellianism plays a core predictive role in MD, boasting the highest O-EI and B-EI across time points, with strong links to multiple MD strategies. Psychopathy ranks second in influence, with its predictive focus shifting from displacement of responsibility to advantageous comparison as adolescents’ cognition develops. In contrast, narcissism exerts minimal, even negative, predictive effects on MD. Notably, MD exerts a modest shaping effect on DT traits, particularly on Machiavellianism and psychopathy. Gender-based comparisons show distinct network structures, with Machiavellianism as the key bridge between DT and MD in males and psychopathy fulfilling this role in females. These findings deepen the mechanistic understanding of longitudinal, reciprocal, and gender-differentiated DT–MD dynamics in adolescence and provide empirically supported guidance for the development of targeted ethical education and gender-specific intervention programs.

## Figures and Tables

**Table 1:** Item labels and descriptive statistics for the three time points.

Item	Label	Time 1		Time 2		Time 3	
		*M*	*SD*	*M*	*SD*	*M*	*SD*
Machiavellianism	M	2.73	1.52	3.11	1.56	3.20	1.41
Psychopathy	N	2.58	1.38	2.93	1.47	3.03	1.39
Narcissism	P	4.21	1.48	4.06	1.45	4.00	1.29
Moral Justification	MD1	3.27	1.84	3.21	1.74	3.29	1.63
Euphemistic Labelling	MD2	2.24	1.57	2.64	1.67	2.77	1.55
Advantageous Comparison	MD3	1.75	1.41	2.43	1.74	2.60	1.63
Displacement of Responsibility	MD4	2.99	1.82	3.19	1.75	3.27	1.59
Diffusion of Responsibility	MD5	3.45	1.87	3.53	1.78	3.57	1.66
Distortion of Consequences	MD6	1.88	1.46	2.48	1.74	2.68	1.64
Attribution of Blame	MD7	3.28	1.96	3.31	1.89	3.26	1.67
Dehumanization	MD8	2.95	1.76	3.18	1.78	3.29	1.59

## Data Availability

The data that support the findings of this study are available upon request from the corresponding author.

## Supplementary Materials

The following supporting information can be downloaded at https://www.mdpi.com/article/10.3390/bs16030398/s1. Table S1: The T1→T2 edge weight matrix; Table S2: The T2→T3 edge weight matrix; Figure S1: Accuracy of T1→T2 network edge weight; Figure S2: Accuracy of T2→T3 network edge weight; Figure S3: Stability of T1→T2 network centrality; Figure S4: Stability of T2→T3 network centrality; Figure S5: Stability of males T1→T2 network centrality; Figure S6: Stability of males T2→T3 network centrality; Figure S7: Stability of females T1→T2 network centrality; Figure S8: Stability of females T2→T3 network centrality.

## Abbreviations

The following abbreviations are used in this manuscript:

MD	Moral disengagement
DT	Dark Triad
CLPN	Cross-Lagged Panel Network
MD1	Moral justification
MD2	Euphemistic labelling
MD3	Advantageous comparison
MD4	Displacement of responsibility
MD5	Diffusion of responsibility
MD6	Distortion of consequences
MD7	Attribution of blame
MD8	Dehumanization
O-EI	Out Expected Influence
I-EI	In Expected Influence
B-EI	Bridge Expected Influence
CIs	Confidence Intervals
