# Supplementary material for: The Longitudinal Relationship Between Dark Triad Traits and Moral Disengagement in Adolescents: A Cross-Lagged Panel Network Analysis
Source: Behav Sci (Basel). 2026 Mar 9;16(3):398. doi: 10.3390/bs16030398 (PMC13024257; doi:10.3390/bs16030398)
Supplement: Supplementary file 1 [file behavsci-16-00398-s001.zip › behavsci-4152679-supplementary.pdf]

The longitudinal relationship between Dark Triad traits and moral disengagement in adolescents: A cross-lagged panel network analysis

Supplementary Materials

Table S1. The T1→T2 edge weight matrix

	MD1	MD2	MD3	MD4	MD5	MD6	MD7	MD8	M	P	N
MD1	0	0.09	0.06	0.09	0.04	0.06	0.04	0.05	0.08	0.06	0.06
MD2	0.01	0	0.08	0	0	0.11	0.03	0	0.01	0.02	0
MD3	0	0.02	0	0	0	0.03	0	–0.03	0	0	–0.01
MD4	0.03	0	0	0	0.05	0	0.04	0.06	0	0.01	0.05
MD5	–0.03	–0.04	–0.04	0.01	0	–0.05	0	0	0	0	–0.01
MD6	0.03	0.1	0.11	0.01	0	0	0	0	0	0.03	–0.04
MD7	0	–0.02	0	0	0	–0.02	0	0.03	0	0	–0.02
MD8	0.06	0	0	0	0.02	0	0.02	0	0	0	0.05
M	0.07	0.12	0.12	0.02	0.04	0.13	0.07	0.08	0	0.09	0.05
P	0	0.03	0.07	0.13	0.05	0.06	0.09	0.12	0.07	0	–0.02
N	0	0	0	0	0	–0.01	–0.06	–0.02	0	0	0

Note. MD1: Moral Justification; MD2: Euphemistic Labelling; MD3: Advantageous Comparison; MD4: Displacement of Responsibility; MD5: Diffusion of Responsibility; MD6: Distortion of Consequences; MD7: Attribution of Blame; MD8: Dehumanization; M: Machiavellism; P: Psychopathy; N: Narcissism.

Table S2. The T2→T3 edge weight matrix

	MD1	MD2	MD3	MD4	MD5	MD6	MD7	MD8	M	P	N
MD1	0	0.01	0	0	0.04	0	0.04	0.04	0	0	–0.04
MD2	–0.08	0	0.04	0	0	0	0.03	0	0	0	–0.07
MD3	–0.06	0	0	0	–0.06	0.02	–0.07	–0.02	0.02	0.01	0
MD4	0.04	0	0	0	0.04	0	0	0	0	0.01	0.07
MD5	–0.04	0	0	0.04	0	–0.07	0	0	0	0	0
MD6	0.05	0.11	0.13	0.03	–0.01	0	0.01	0	0.01	0.02	0.04
MD7	–0.05	0	0	–0.01	–0.04	0	0	0.01	0	0	–0.04
MD8	0.06	0.04	0.05	0.05	0.03	0.06	0.09	0	0.09	0.02	0
M	0.10	0.05	0.06	0.09	0.06	0.15	0.13	0.14	0	0.15	0.12
P	0.08	0.08	0.17	0.01	0.04	0.10	0	0	0.02	0	–0.13
N	0	0	0	0.07	0.09	–0.02	0.02	0.05	0	0	0

Note. MD1: Moral Justification; MD2: Euphemistic Labelling; MD3: Advantageous Comparison; MD4: Displacement of Responsibility; MD5: Diffusion of Responsibility; MD6: Distortion of Consequences; MD7: Attribution of Blame; MD8: Dehumanization; M: Machiavellism; P: Psychopathy; N: Narcissism.

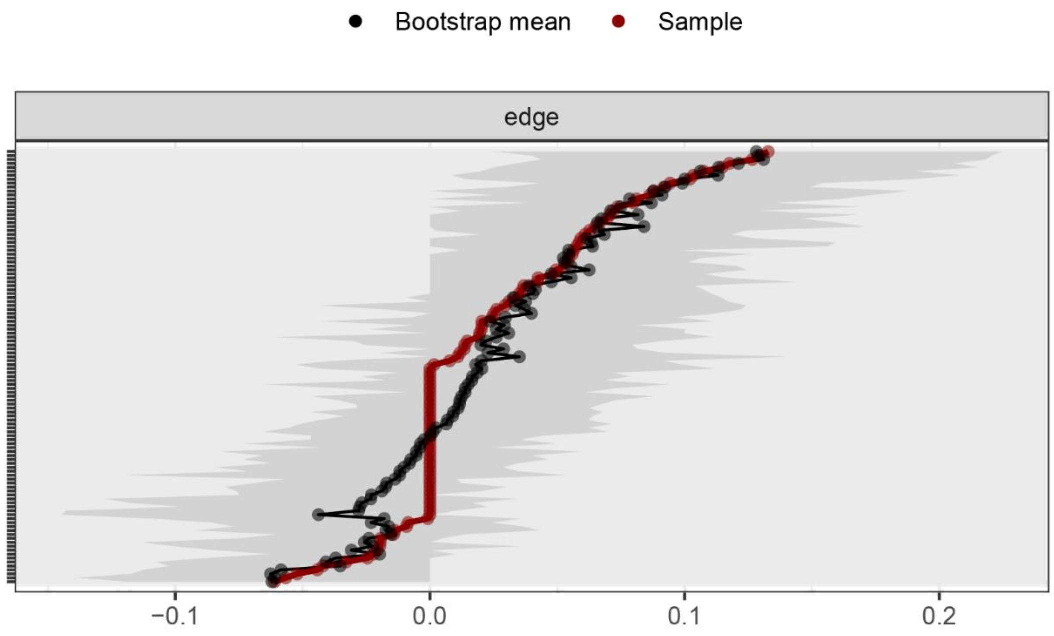

Figure S1. Accuracy of T1→T2 network edge weight

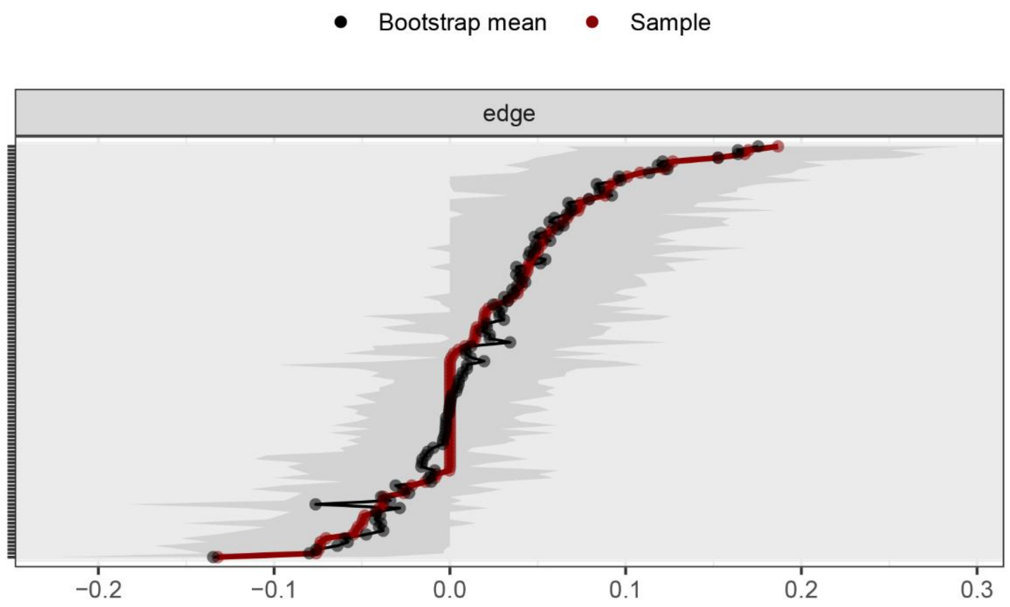

Figure S2. Accuracy of T2→T3 network edge weight

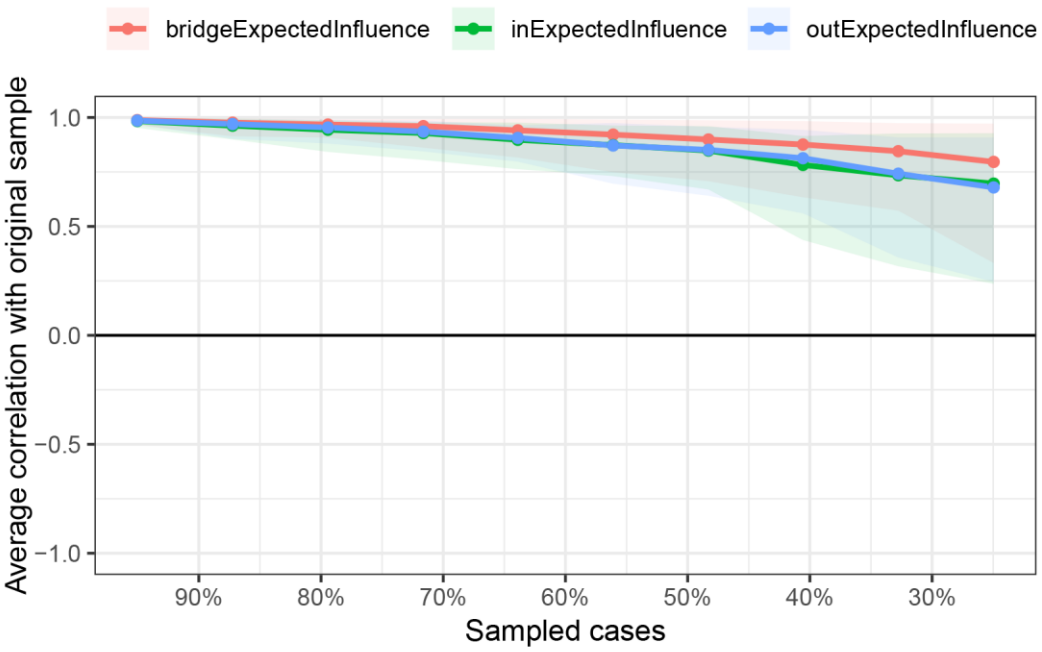

Figure S3. Stability of T1→T2 network centrality

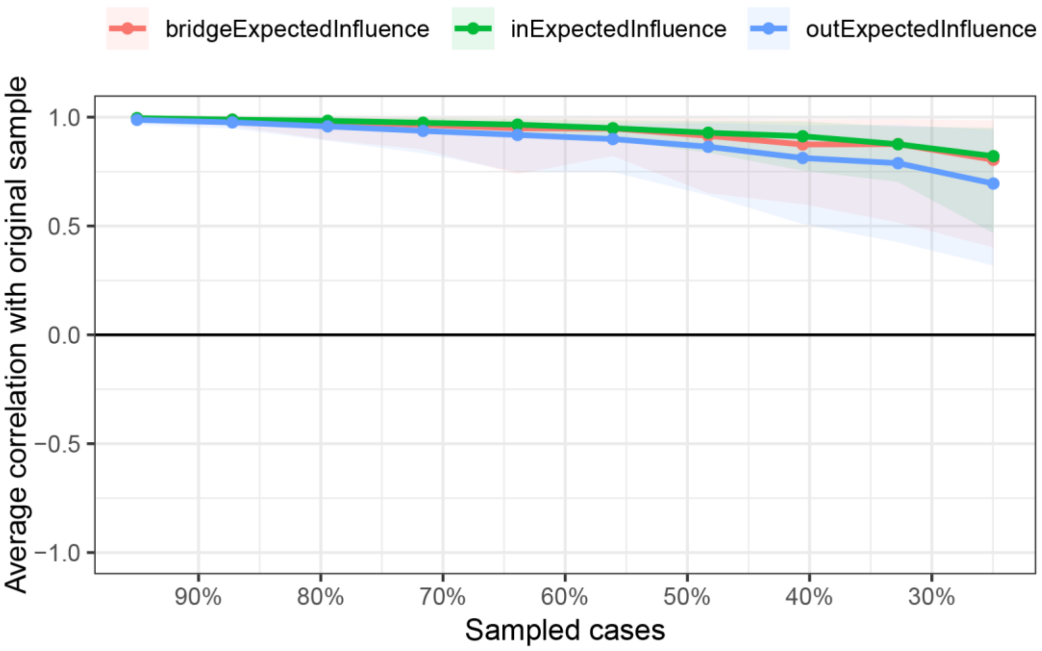

Figure S4. Stability of T2→T3 network centrality

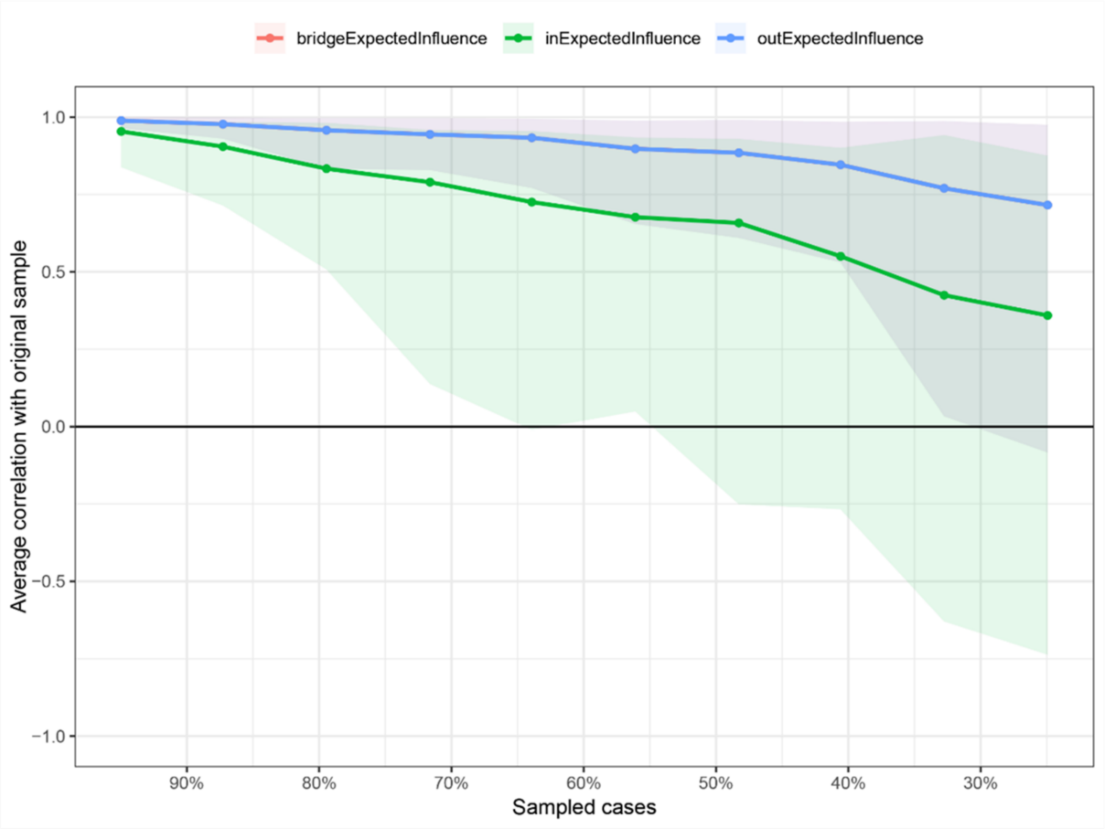

Figure S5. Stability of males T1→T2 network centrality

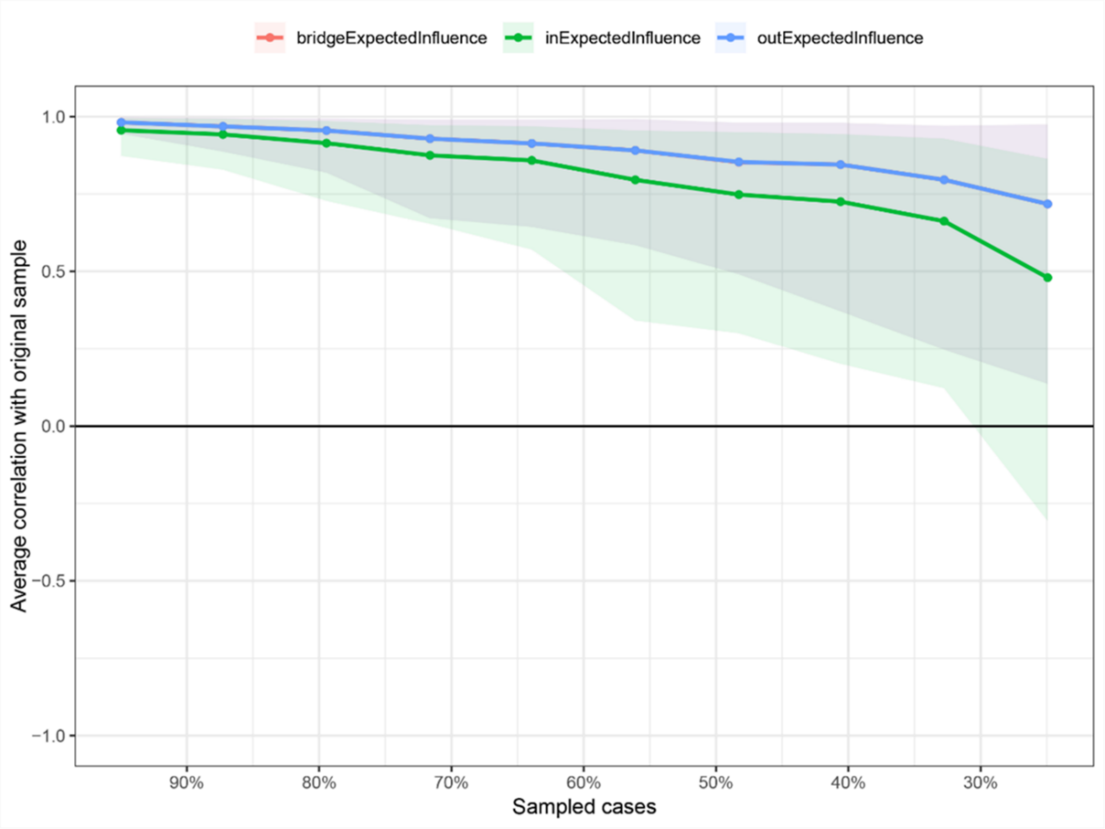

Figure S6. Stability of males T2→T3 network centrality

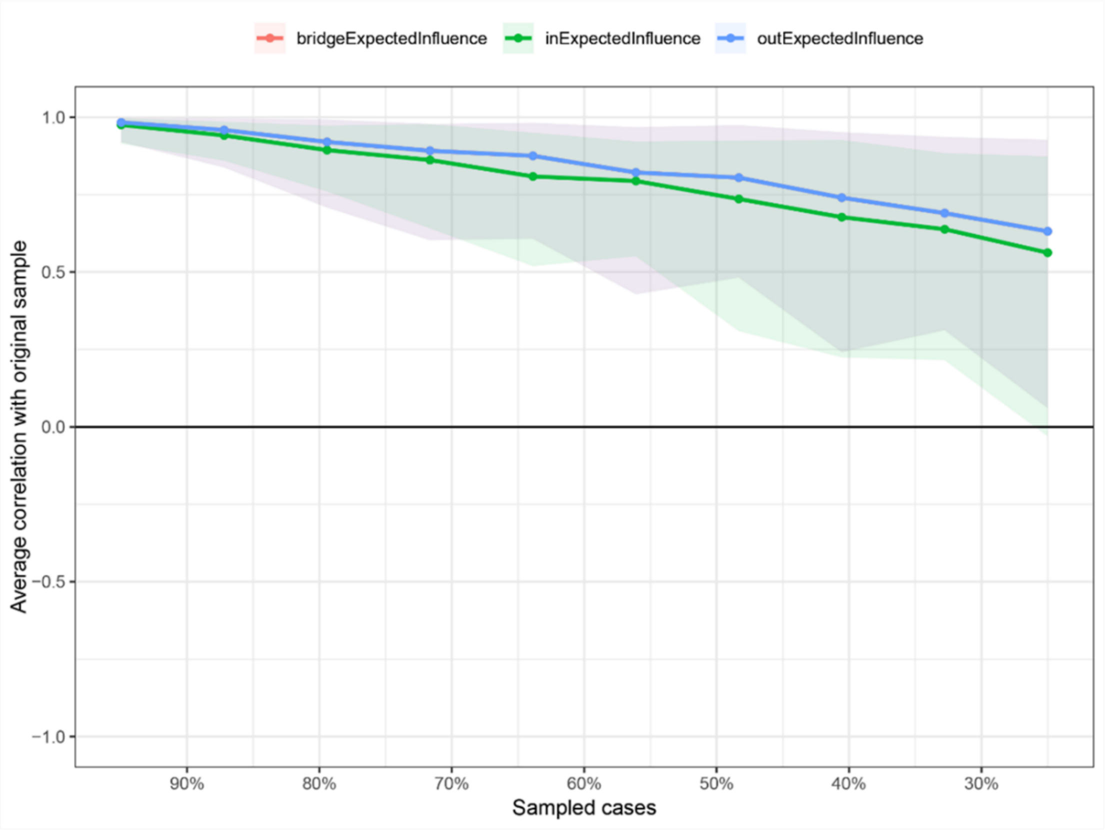

Figure S7. Stability of females T1→T2 network centrality

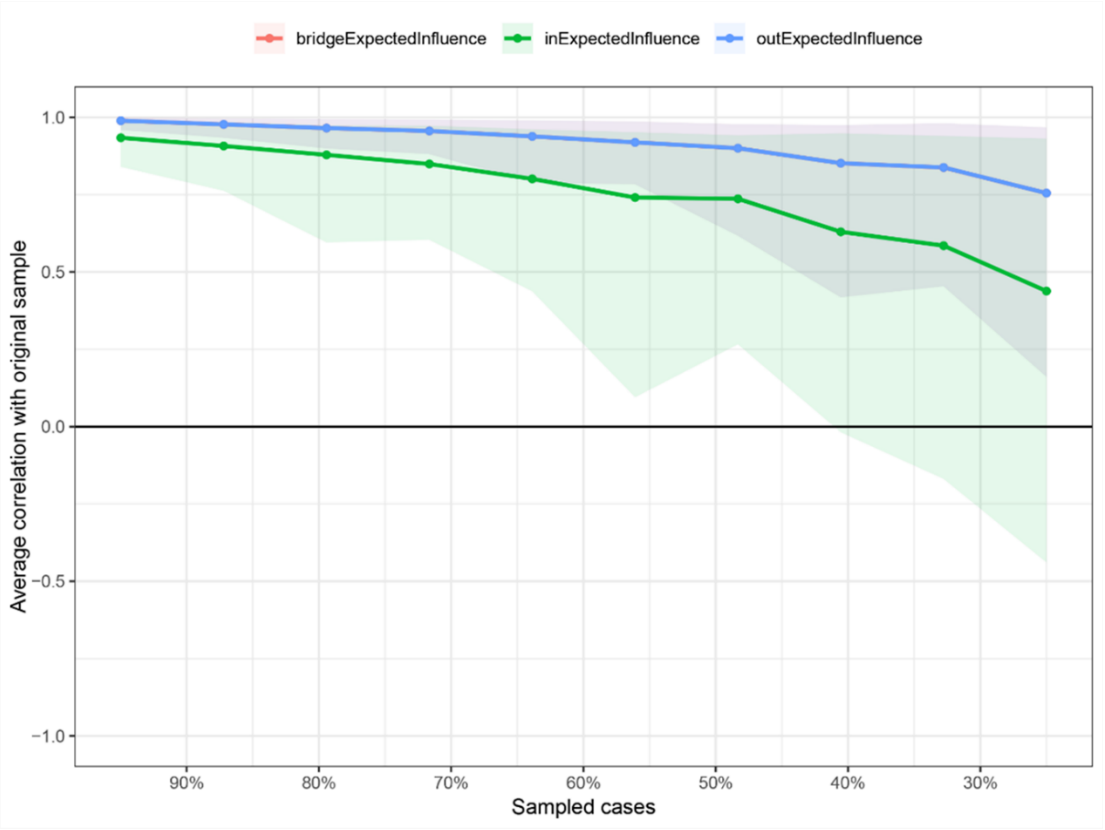

Figure S8. Stability of females T2→T3 network centrality
